# Haplotype-GGGT in long non-coding RNA MALAT1 inhibits brain metastatic lung cancer and lymph nodes of lung cancer via the MALAT1/miR-328/KATNB1

**DOI:** 10.18632/aging.204563

**Published:** 2023-03-02

**Authors:** Tingting Liu, Jianpeng Ma, Dongmei Hou, Weiqi Wang, Hetao Cao

**Affiliations:** 1 Department of Medical Imaging, Affiliated Hospital of Nantong University, Nantong, Jiangsu 226001, China; 2 Department of Magnetic Resonance Imaging, Dingbian County People’s Hospital, Dingbian, Yulin, Shaanxi 718600, China; 3 School of Pharmacy, Nantong University, Nantong, Jiangsu 226001, China

**Keywords:** lung cancer, miRNA, brain metastatic lung cancer, MALAT1, katanin P80, miR-328

## Abstract

The up-regulation of katanin P80 has been reported to be correlated with a larger tumor size and lymph node metastasis in non–small-cell lung cancer (NSCLC) patients. And lncRNA MALAT1 was demonstrated to promote the proliferation of chronic myeloid leukemia cells via modulating miR-328. 135 lung cancer patients were divided into 6 groups according to their genotypes of MALAT1. The expression of KATNB1 was negatively correlated with the GGGT genotype of MALAT1. Decreased lymph node size and tumor size of brain metastatic lung were observed in patients with GGGT genotype of MALAT1. The luciferase activities of MALAT1 and KATNB1 were remarkably suppressed by miR-328 in A549 and H460. And the down-regulation of MALAT1 or up-regulation of miR-328 significantly repressed the KATNB1 expression in A549 and H460 cells. MALAT1 expression was reduced in patients carrying haplotype GGGT. A signaling pathway of MALAT1/miR-328/KATNB1 was established to explain the down-regulation of KATNB1 mRNA in patients carrying haplotype GGGT and reduced lymph node size in lung cancer and tumor size in brain metastatic lung cancer.

## INTRODUCTION

As the leading cause of mortality in both women and men, lung cancer has poor prognosis [1]. In addition to the site of the primary cancer and the ability of cancer cell infiltration to neighboring tissues, the condition of lymph nodes is also important in the pre-therapeutic stage of lung cancer. Metastasis to mediastinal lymph nodes reduces the rate of survival, while patients with metastasis to contralateral hilar and contralateral mediastinal lymph nodes are less resectable [2]. Metastasis of cancer cells to distant tissues is a major cause of cancer-related deaths [3–5]. Nevertheless, although many mechanistic studies have helped scientists to gain insight in the process of metastasis, the epidemiology of metastasis in cancer remains elusive.

Many studies have shown that miR-328 participate in various cellular processes of cancer cells by acting as either a tumor suppressor or an oncogene, thus suggesting miR-328 to be tumor- as well as tissue-specific. Moreover, colon cancer stem cells (CSCs) were shown to have low expression of miR-328, suggesting that the downregulation of miR-328 expression can aid the development of CSCs in the colorectal cancer [6].

As one of most abundant and conserved lncRNAs, the metastasis associated lung adenocarcinoma transcript 1 (MALAT1) was shown to mediate alternative splicing of pre-mRNAs [7]. Nonetheless, MALAT1-knockout mice displayed no phenotypic variations, while the ablation of the MALAT1 gene did not change the global level of gene expression and pre-mRNA splicing [8–10]. Besides, MALAT1 may also act as a sponge of miRNAs to reduce miR-328 activity. For example, the knockdown of MALAT1 boosted miR-328 expression, whereas the upregulation of MALAT1 inhibited the expression of miR-328 [11].

Previous study showed that the rs619586-A and rs3200401-T haplotype of MALAT1 is associated with a higher risk of multiple sclerosis (MS), providing additional evidence for the role of MALAT1 in MS pathogenesis [12]. The high expression of katanin P80, which is encoded by KATNB1 mRNA, has been reported to be correlated with a larger tumor size and lymph node metastasis in non–small-cell lung cancer (NSCLC) patients [13]. Also, lncRNA MALAT1 was demonstrated to promote the proliferation of chronic myeloid leukemia cells via modulating miR-328 [11]. In this study, by studying a group of lung cancer patients, we aimed to investigate the association between different haplotypes of MALAT1 and the tumor size of in the lymph node of lung cancer and brain metastatic lung cancer.

## MATERIALS AND METHODS

### Human sample collection

**Figure 1:**
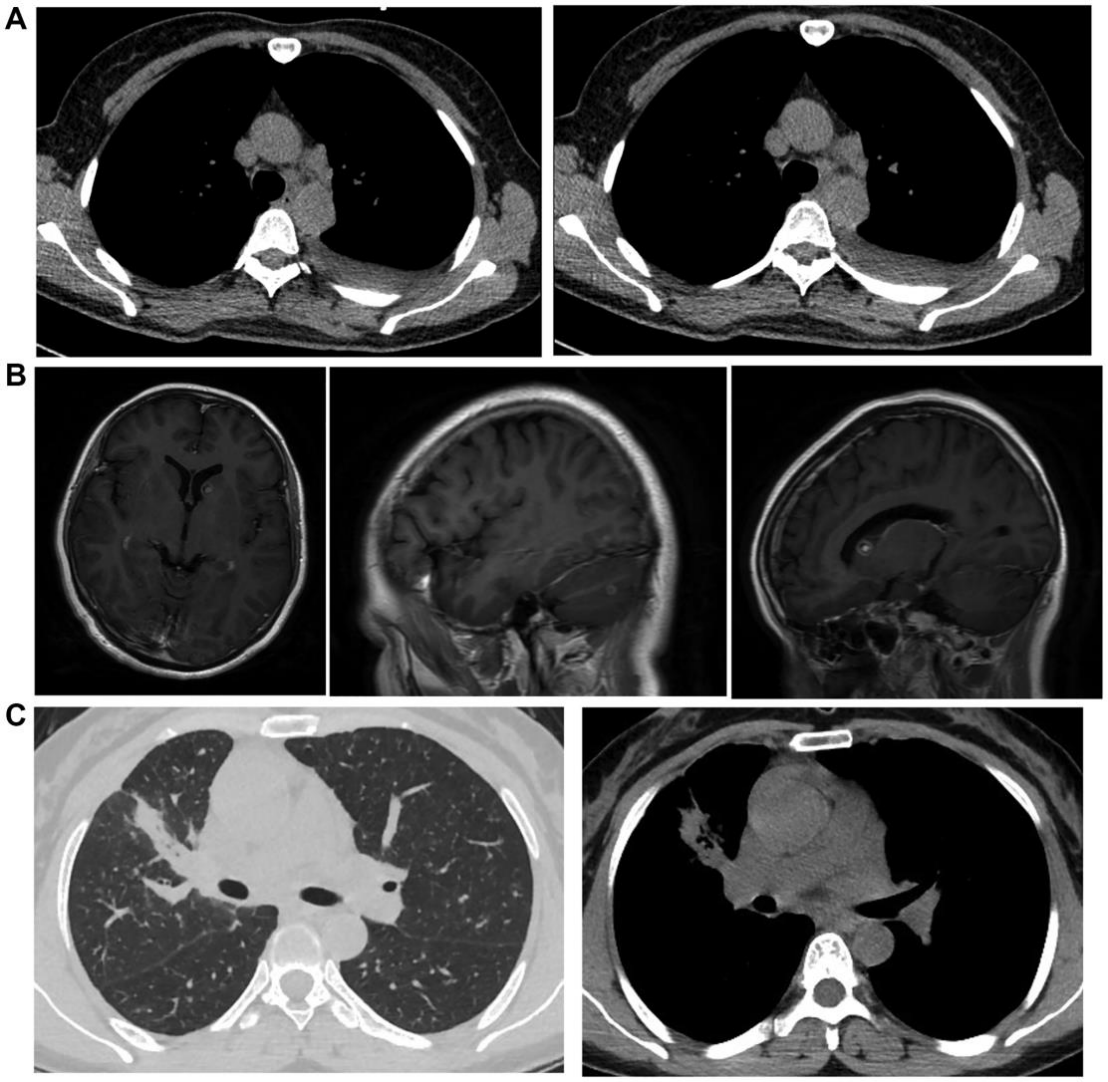
**The MRI images of metastatic lung cancer patients.** (**A**) MRI image of mediastinal lymph node metastasis and left side pleural effusion. (**B**) MRI image of brain metastatic non-small cell lung cancer. (**C**) MRI image of non-small cell lung cancer in the upper lobe of the right lung with metastasis in both lungs and mediastinum.

This study recruited 135 patients with brain metastatic lung cancer who were diagnosed with magnetic resonance imaging (MRI) technology (Figure 1) and collected the tissue samples from all of the patients. Genotyping was carried out to determine the genotypes of rs11227209, rs619586, rs664589 and rs3200401 located in MALAT1. The patients were grouped according to their genotypes of MALAT1: 1. CACC (*N* = 65), 2. GGGT (*N* = 28), 3. CGCC (*N* = 16), 4. CAGC (*N* = 7), 5. GAGT (*N* = 3), 6. CA (*N* = 12). The basic characteristics including age at diagnosis, gender, cigarette use history, primary disease history and overall stage of tumor of the patients in the six groups were collected. Then, among all variables, continuous variables were expressed in median, while discrete variables were expressed in the number of cases and percentage. In order to find variables that are linked to the incidence of asymptomatic brain metastasis, SPSS 22.0 (Version number 22.0, IBM, SPSS Inc., Chicago, IL, USA) was utilized. In addition, univariate as well as multivariate logistic regressions were carried out to evaluate the effects of subsequent factors: History of cigarette smoking, largest size of the mediastinal lymph node, and the optimal dissection point for the largest lymph node. Finally, peripheral blood and lung cancer tissue samples were collected from the patients to analyze the expression of target genes. Institutional ethical committee has approved the protocol of this study. Written informed consent was obtained from each patient before the initiation of this study.

### Real-time PCR

Real-time PCR was carried out to assess the expressions of MALAT1, miR-328, and KATNB1 in each sample. First, total RNA was extracted from the samples using a Trizol reagent or a mirVana Paris Isolation assay kit (Thermo Fisher Scientific, Waltham, MA, USA) for MALAT1/miR-328 and KATNB1, respectively, according to the recommended protocols provided by the product manuals of assay kit manufacturers. The quality as well as concentration of isolated RNA was assayed by using the Nano Drop 2000 Spectrophotometer (Thermo Fisher Scientific, Waltham, MA, USA) and evaluated via the OD260/OD280 ratio. In the next step, the isolated RNA of KATNB1 was subject to reverse transcription carried out using the TaqMan MicroRNA RT kit (Thermo Fisher Scientific, Waltham, MA, USA) according to the recommended protocol provided by the product manual of assay kit manufacturer, while the isolated MALAT1/miR-328 was directly evaluated by using a Taqman miRNA assay (Thermo Fisher Scientific, Waltham, MA, USA) according to the recommended protocol provided by the product manual of assay kit manufacturer. The real time PCR was carried out on an ABI 7900HT system (ABI, Foster City, CA, USA). The relative expression of MALAT1 (Primer sequences: F-GAATTGCGTCATTTAAAGCCTAGTT; R-GTTTCATCCTACCACTCCCAATTAAT), miR-328 (Primer sequences: F- CCTCTCTGCCCTTCCG; R- GAACATGTCTGCGTATCTC), and KATNB1 (Primer sequences: F-GCCACAAGAACCTGGACACTGT; R- CTGGTTGACGATGTTCAGGAGG) in each sample was calculated by using the 2^−ΔΔCt^ approach, using GAPDH and U6 as the internal reference genes.

### Cell model establishment

A549/H460 cells (purchased from Cell Resource Center of the Shanghai Institute of Biochemistry, Shanghai, China) were cultured under the vendor-recommended conditions: Saturated humidity, 37^o^C and 5 % carbon dioxide in DMEM (Hyclone, GE Health Life Sciences, Piscataway, NJ, USA) supplemented with 10 % fetal bovine serum (Hyclone, GE Health Life Sciences, Piscataway, NJ, USA) and antibiotics. On the day of experiments, the A549/H460 cells were used to establish 2 cellular models: In cellular model I, A549/H460 cells were divided into 3 groups, i.e., 1. NC group (A549/H460 cells transfected with a negative control siRNA); 2. MALAT1 siRNA group (A549/H460 cells transfected with MALAT1 siRNA); and 3. miR-328 precursor group (A549/H460 cells transfected with miR-328 precursors). In cellular model II, A549/H460 cells were also divided into 3 groups, i.e., 1. NC group (A549/H460 cells transfected with a negative control empty plasmid); 2. P-MALAT1 group (A549/H460 cells transfected with the plasmid carrying the MALAT1 gene); and 3. P-anti-miR-328 group (A549/H460 cells transfected with the plasmid carrying anti-miR-328). All transfection was carried out by using Lipofectamine 3000 (Invitrogen, Carlsbad, CA, USA) according to the recommended transfection protocol provided by the product manual of transfection reagent manufacturer, and all transfected cells at a density of 10^5^/well in a 24-well plate were harvested 24 h post transfection to assess the expression of target genes.

### Vector construction and luciferase assay

Our initial sequence analysis showed that miR-328 could potentially target MALAT1 and the 3′UTR of KATNB1. In order to explore the regulatory relationship between MALAT1 and miR-328 as well as between KATNB1 and miR-328, luciferase assays were performed. In brief, wild type vectors containing wild type MALAT1 and KATNB1 3′UTR sequences containing the potential miR-328 binding sites were established by inserting corresponding sequences into pcDNA luciferase vectors (Promega, Madison, WI, USA), respectively. At the same time, site directed mutagenesis was carried out by using the Quick Change mutagenesis assay kit (Stratagene, San Diego, CA, USA) according to the recommended protocol provided by the product manual of assay kit manufacturer to create single mutations in the miR-328 binding sites of wild type MALAT1 and KATNB1 3′UTR sequences, followed by the insertion of mutant sequences into pcDNA luciferase vectors to establish mutant type vectors of MALAT1 and KATNB1 3′UTR. In the next step, A549 and H460 cells were co-transfected with wild type or mutant type MALAT1 and KATNB1 3′UTR vectors along with miR-328 or a control miRNA using Lipofectamine 3000 (Invitrogen, Carlsbad, CA, USA) according to the recommended transfection protocol provided by the product manual of transfection reagent manufacturer, and the luciferase activities of transfected A549 and H460 cells were measured 48 h later by using a Bright-Glo luciferase assay system (Promega, Madison, WI, USA) according to the recommended protocol provided by the product manual of assay kit manufacturer.

### Statistical analysis

In this study, continuous variables were expressed in median, while discrete variables were expressed in the number of cases and percentage. All statistical analyses were carried out using SPSS 22.0 (Version number 22.0, IBM, SPSS Inc., Chicago, IL, USA). A *p* value of < 0.05 indicated statistical significance.

### Availability of data and material

The data of this study are available from the corresponding author upon reasonable request.

## RESULTS

### Patient characteristics

This study recruited 135 patients with brain metastatic lung cancer and collected the tissue samples from all of the patients. The basic characteristics including age at diagnosis, gender, cigarette use history, primary disease history and overall stage of tumor of the patients were compared among the six groups, indicating insignificant differences in respect to these parameters listed (Table 1).

**Table 1:** The basic characteristics of recruited brain metastatic lung cancer patients.

**Characteristics**	**CACC (*N* = 65)**	**GGGT (*N* = 28)**	**CGCC (*N* = 16)**	**CAGC (*N* = 7)**	**GAGT (*N* = 3)**	**CACT (*N* = 12)**	***P* value**
Sex, male	40 (61.5)	18 (64.3)	8 (50.0)	4 (57.1)	2 (66.7)	8 (66.7)	0.111
Median age at diagnosis, years	64.1 ± 5.9	63.5 ± 4.8	61.5 ± 6.6	66.5 ± 7.5	61.2 ± 5.3	60.8 ± 5.2	0.760
Cigarette use							
Never-smoker	8 (12.3)	5 (17.9)	3 (18.8)	1 (14.3)	0 (0)	1 (8.3)	0.456
Current smoker	57 (87.7)	23 (82.1)	13 (81.2)	6 (85.7)	3 (100.0)	11 (91.7)	
Primary histology							
Adenocarcinoma	33 (50.8)	18 (64.3)	7 (43.8)	4 (57.1)	2 (66.7)	5 (41.7)	0.420
SCC	20 (30.8)	8 (28.6)	4 (25.0)	2 (28.6)	1 (33.3)	3 (25.0)	
Large-cell	8 (12.3)	2 (10.7)	2 (12.5)	1 (14.3)	0 (0)	2 (16.7)	
NOS	4 (6.1)	0 (0)	3 (18.7)	0 (0)	0 (0)	2 (16.6)	
Overall stage							
I	23 (35.4)	8 (28.6)	6 (37.5)	2 (28.6)	1 (33.3)	3 (25.0)	0.836
II	20 (30.8)	8 (28.6)	4 (25.0)	2 (28.6)	1 (33.3)	4 (33.3)	
IIIA	20 (30.8)	10 (35.7)	6 (37.5)	3 (42.8)	1 (33.3)	5 (41.7)	
IIIB	2 (3.0)	2 (7.1)	1 (0)	0 (0)	0 (0)	0 (0)	

### GGGT genotype of MALAT1 was associated with suppressed MALAT1 expression and elevated miR-328 expression in the peripheral blood of lung cancer patients

**Figure 2:**
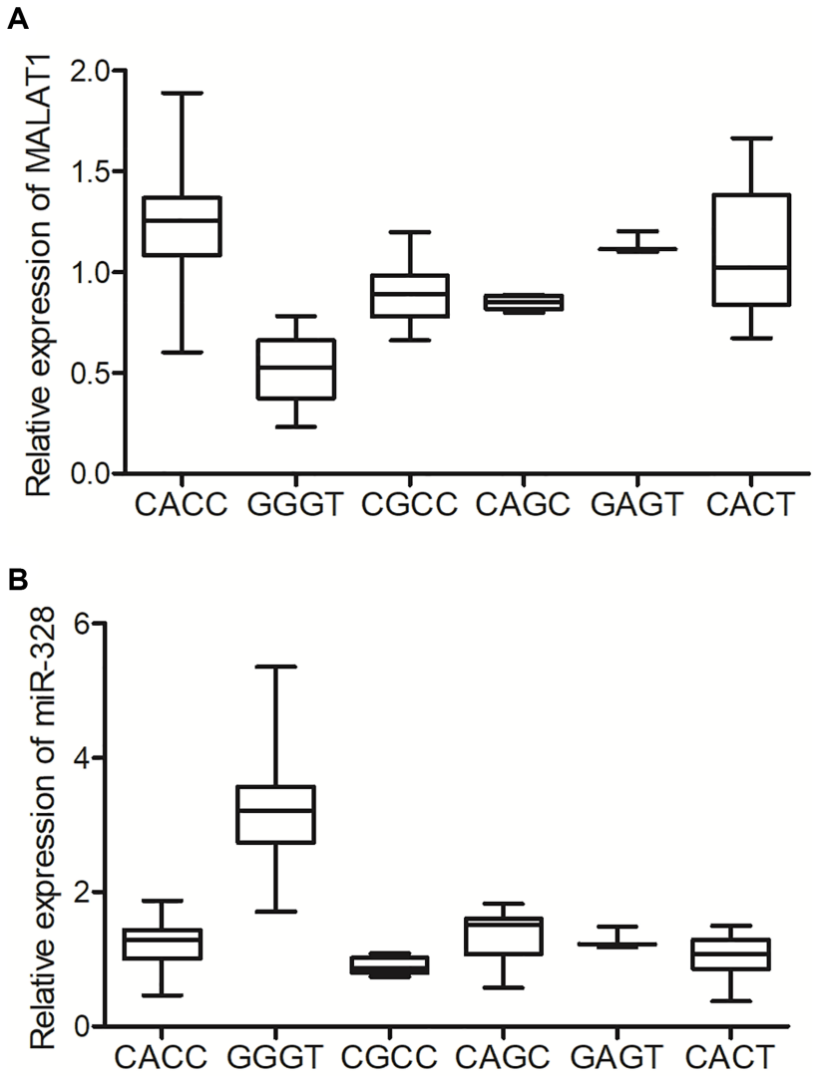
**The GGGT genotype of MALAT1 was correlated with suppressed expression of MALAT1 and elevated expression of miR-328 in the peripheral blood of lung cancer patients.** (**A**) The expression of MALAT1 was suppressed in the peripheral blood of lung cancer patients carrying the GGGT genotype of MALAT1. (**B**) The expression of miR-328 was activated in the peripheral blood of lung cancer patients carrying the GGGT genotype of MALAT1.

Peripheral blood samples were harvested from patients with different genotypes at rs11227209, rs619586, rs664589 and rs3200401 of MALAT1. Quantitative real-time PCR was performed to examine the expression of MALAT1 and miR-328 in the peripheral blood samples collected from lung cancer patients with different MALAT1 genotypes. The expression of MALAT1 was significantly decreased in the peripheral blood of patients with GGGT genotype when compared with patients with CACC, CGCC, CAGC, GAGT and CA genotypes (Figure 2A). On the contrary, the expression of miR-328 in the peripheral blood of patients with GGGT genotype was remarkably elevated in comparison to patients with other genotypes (Figure 2B).

### GGGT genotype of MALAT1 was associated with suppressed MALAT1/KATNB1 expression and elevated miR-328 expression in the cancer tissue samples of lung cancer patients

**Figure 3:**
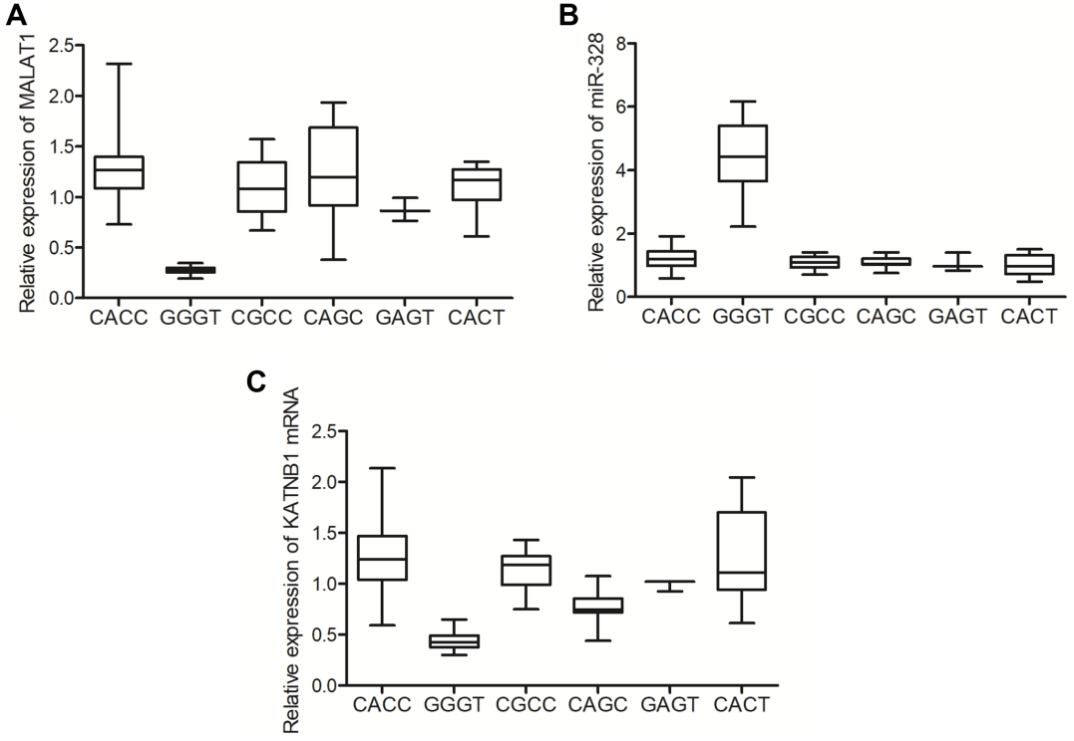
**The GGGT genotype of MALAT1 was correlated with suppressed expression of MALAT1 and elevated expression of miR-328 in the tissue samples of lung cancer patients.** (**A**) The expression of MALAT1 was suppressed in the tissue samples of lung cancer patients carrying the GGGT genotype of MALAT1. (**B**) The expression of miR-328 was activated in the tissue samples of lung cancer patients carrying the GGGT genotype of MALAT1. (**C**) The expression of KATNB1 mRNA was suppressed in the tissue samples of lung cancer patients carrying the GGGT genotype of MALAT1.

In order to directly assess the effect of MALAT1 genotypes on the expression of MALAT1 and miR-328 in lung cancer patients, carcinoma lung tissue samples were harvested from lung cancer patients to evaluate MALAT1 and miR-328 expression. The expression of MALAT1 (Figure 3A) in the tissue samples from patients with GGGT genotype was notably repressed, while the expression of miR-328 (Figure 3B) was apparently increased in the tissue samples of patients with GGGT genotype. Besides, the expression of KATNB1 mRNA and protein was evaluated as shown by qPCR and immunohistochemistry, respectively. The expression of KATNB1 mRNA (Figure 3C) was notably suppressed in the tissue samples collected from patients with the GGGT genotype when compared with patients of CACC, CGCC, CAGC, GAGT and CA genotypes.

### GGGT genotype of MALAT1 was associated with decreased size of lymph node in lung cancer and brain metastatic lung tumor

**Figure 4:**
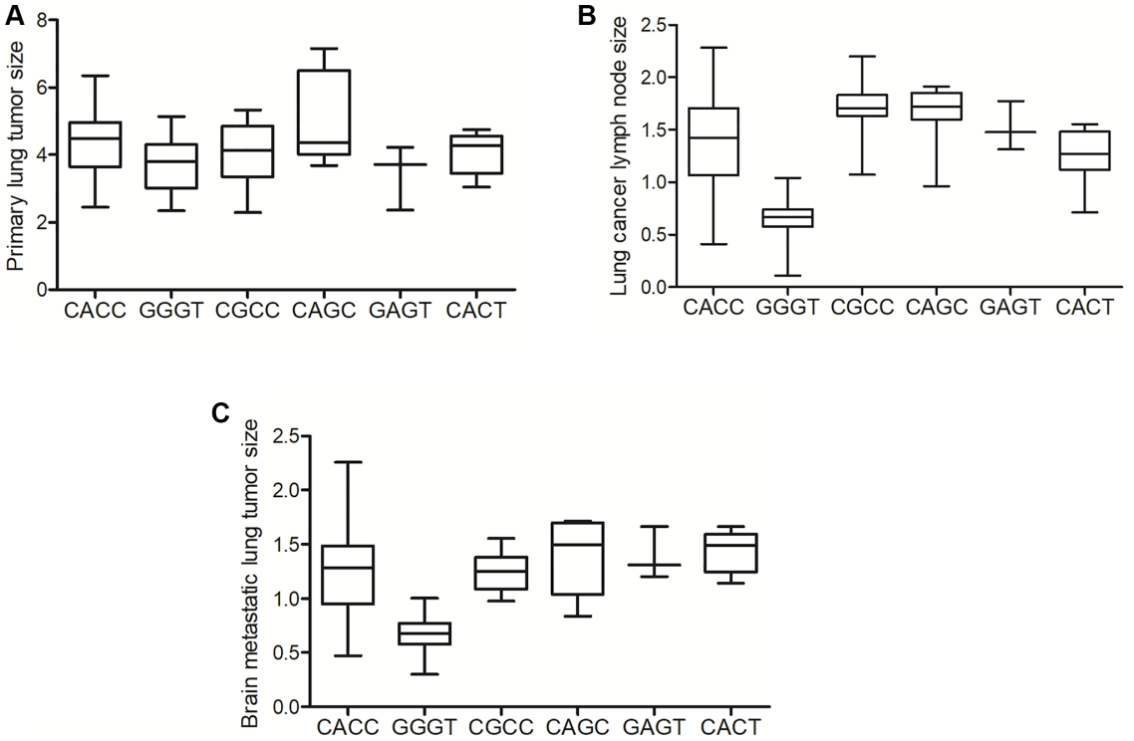
**The GGGT genotype of MALAT1 was correlated with the decreased size of lung cancer lymph node and brain metastatic lung tumor.** (**A**) No obvious difference was found in the size of primary lung cancer in patients carrying different genotypes of MALAT1. (**B**) The size of lymph node was remarkably reduced in patients carrying the GGGT genotype of MALAT1. (**C**) The size of brain metastatic lung tumor was remarkably reduced in patients carrying the GGGT genotype of MALAT1.

The size of primary lung tumor was observed, and no obvious difference was found in respect to the size of primary lung cancer in patients with different MALAT1 genotypes (Figure 4A). Furthermore, evaluation was carried out to analyze the size of lymph nodes and brain metastatic lung cancer. Significant decrease in the size of lung cancer lymph nodes (Figure 4B) and brain metastatic lung tumors (Figure 4C) was observed in patients with the GGGT genotype in MALAT1 when compared to patients with CACC, CGCC, CAGC, GAGT and CACT genotypes. Compared with the expression of MALAT1 in patients carrying different genotypes (Figure 3A), it can be indicated that the decreased MALAT1 level is associated with the decreased size of lung cancer lymph nodes (Figure 4B) and brain metastatic lung tumors (Figure 4C).

### MALAT1 and KATNB1 were targeted by miR-328

**Figure 5:**
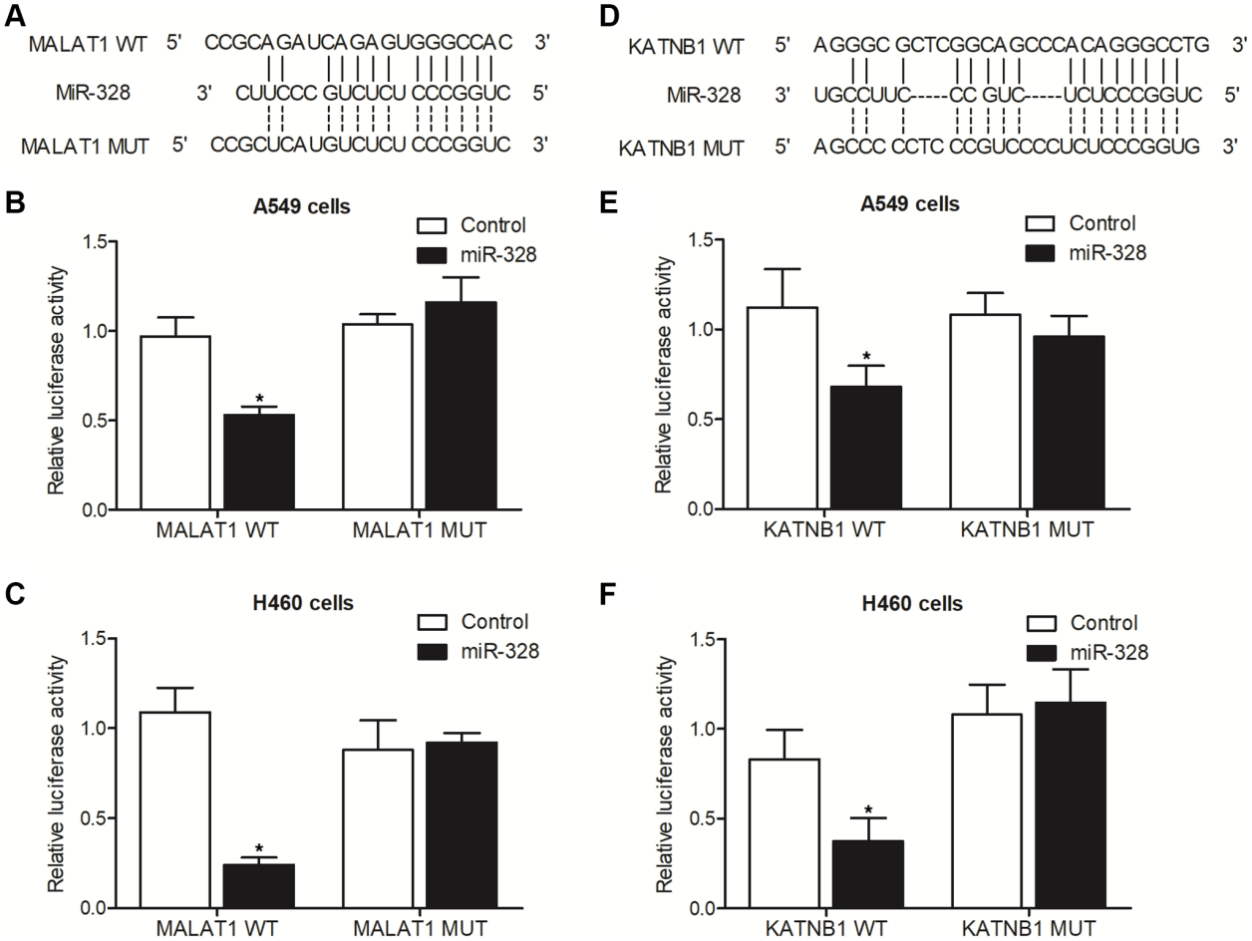
The luciferase activities of MALAT1 and KATNB1 were suppressed by miR-328 in A549 and H460 cells (^*^p value < 0.05 compared with control group). (**A**) Sequence analysis indicated the binding of miR-328 to MALAT1. (**B**) The luciferase activity of wild type MALAT1 was suppressed by miR-328 in A549 cells. (**C**) The luciferase activity of wild type MALAT1 was suppressed by miR-328 in H460 cells. (**D**) Sequence analysis indicated the binding of miR-328 to KATNB1. (**E**) The luciferase activity of wild type KATNB1 was suppressed by miR-328 in A549 cells. (**F**) The luciferase activity of wild type KATNB1 was suppressed by miR-328 in H460 cells.

Sequence analysis showed that miR-328 could potentially target MALAT1 (Figure 5A) and the 3′UTR of KATNB1 (Figure 5D). Luciferase vectors containing wild type and mutant MALAT1 and KATNB1 were established and transfected into A549 and H460 cells along with miR-328. The luciferase activities of wild type MALAT1 were remarkably suppressed in A549 (Figure 5B) and H460 (Figure 5C) cells. Meanwhile, the luciferase activities of wild type KATNB1 were notably repressed in A549 (Figure 5E) and H460 (Figure 5F), whereas the luciferase activities of mutant MALAT1 and KATNB1 remained unchanged.

### Alteration of MALAT1 and miR-328 expression remarkably affected the expression of KATNB1 mRNA and protein in A549 and H460 cells

**Figure 6:**
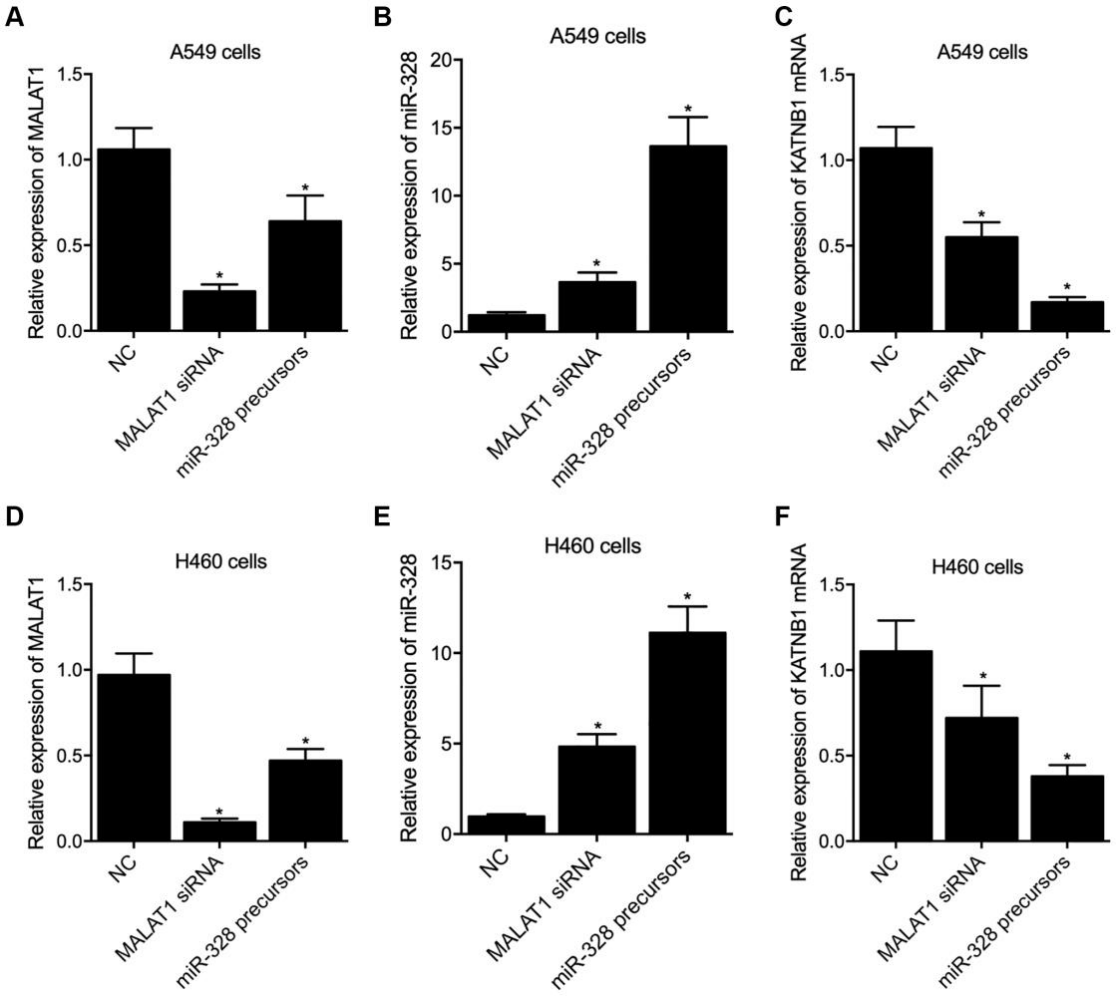
Knockdown of MALAT1 and overexpression of miR-328 suppressed the expression of KATNB1 in A549 and H460 cells (^*^p value < 0.05 compared with NC group). (**A**) The expression of MALAT1 was inhibited by MALAT1 siRNA and miR-328 precursors in A549 cells. (**B**) The expression of miR-328 was activated by MALAT1 siRNA and miR-328 precursors in A549 cells. (**C**) The expression of KATNB1 mRNA was inhibited by MALAT1 siRNA and miR-328 precursors in A549 cells. (**D**) The expression of MALAT1 was inhibited by MALAT1 siRNA and miR-328 precursors in H460 cells. (**E**) The expression of miR-328 was activated by MALAT1 siRNA and miR-328 precursors in H460 cells. (**F**) The expression of KATNB1 mRNA was inhibited by MALAT1 siRNA and miR-328 precursors in H460 cells.

**Figure 7:**
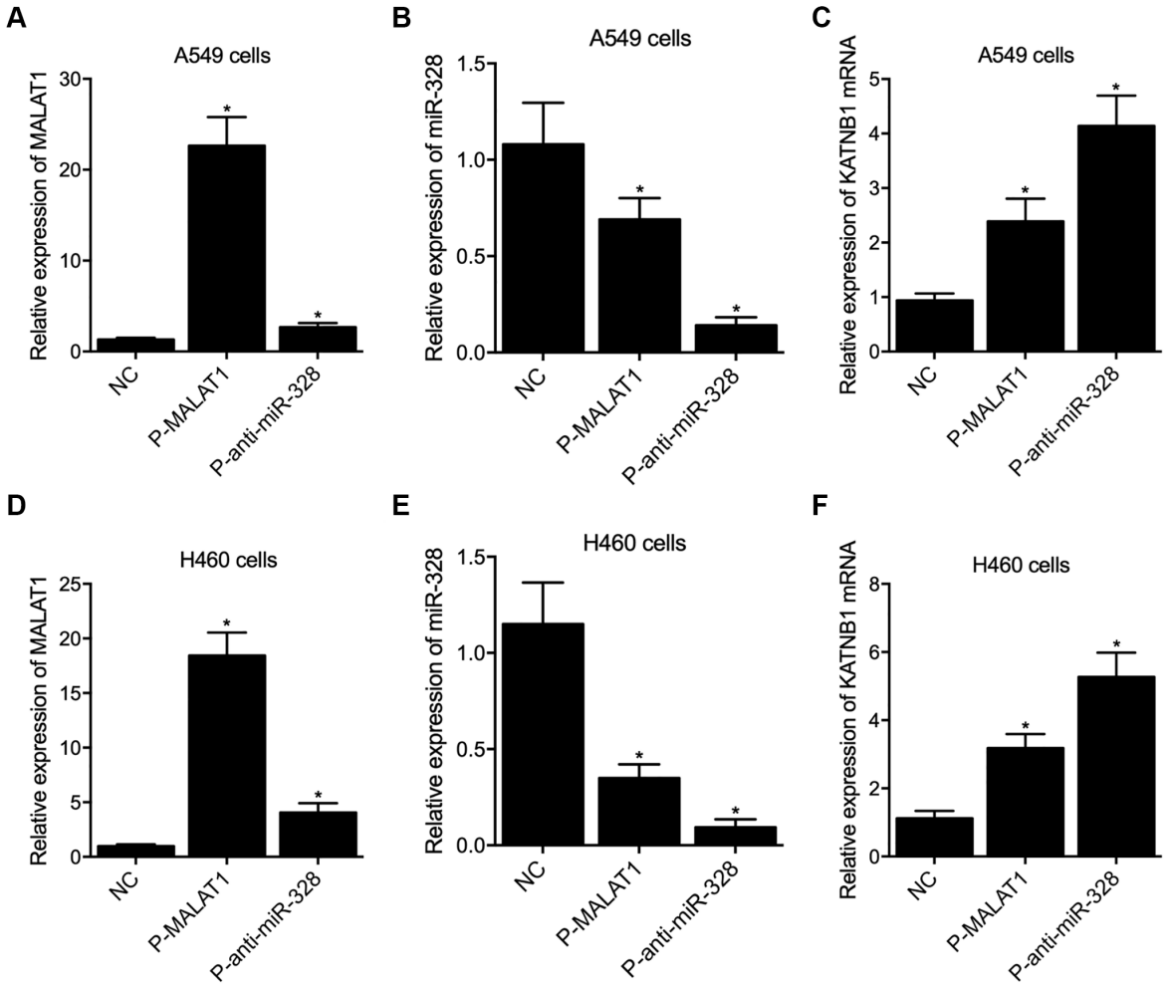
Overexpression of MALAT1 and suppression of miR-328 enhanced the expression of KATNB1 in A549 and H460 cells (^*^p value < 0.05 compared with NC group). (**A**) The expression of MALAT1 was enhanced by P-MALAT1 and P-anti-miR-328 in A549 cells. (**B**) The expression of miR-328 was suppressed by P-MALAT1 and P-anti-miR-328 in A549 cells. (**C**) The expression of KATNB1 mRNA was enhanced by P-MALAT1 and P-anti-miR-328 in A549 cells. (**D**) The expression of MALAT1 was enhanced by P-MALAT1 and P-anti-miR-328 in H460 cells. (**E**) The expression of miR-328 was suppressed by P-MALAT1 and P-anti-miR-328 in H460 cells. (**F**) The expression of KATNB1 mRNA was enhanced by P-MALAT1 and P-anti-miR-328 in H460 cells.

In order to further explore the regulatory network among MALAT1, miR-328 and KATNB1, MALAT1 siRNA and miR-328 precursors were transfected into A549 and H460 cells. Quantitative real-time PCR was performed to examine the expression of MALAT1, miR-328 and KATNB1 in A549 and H460 cells. The expression of MALAT1 was dramatically suppressed in A549 and H460 cells transfected with MALAT1 siRNA, as well as in A549 (Figure 6A) and H460 (Figure 6D) cells transfected with miR-328 precursor. The expression of miR-328 was remarkably enhanced in A549 (Figure 6B) and H460 (Figure 6E) cells transfected with MALAT1 siRNA and miR-328 precursor. The expression of KATNB1 mRNA was apparently repressed in A549 (Figure 6C) and H460 (Figure 6F) cells transfected with MALAT1 siRNA and miR-328 precursor. Meanwhile, transfection of p-MALAT1 and p-anti-miR-328 into A549 and H460 cells obviously enhanced the expression of MALAT1 and suppressed the expression of miR-328, respectively. The expression of KATNB1 mRNA and katanin P80 protein was apparently enhanced in A549 and H460 cells transfected with p-MALAT1 and p-anti-miR-328 (Figure 7).

## DISCUSSION

In this study, we recruited 135 patients with brain metastatic lung cancer and divided them into six groups according to their genotypes of MALAT1. The expression of MALAT1 and miR-328 in the peripheral blood and tissue samples was examined. Obvious suppression of MALAT1 and miR-328 was observed in the peripheral blood and tissue samples collected from patients with the GGGT genotype. MALAT1 is shown to control the growth, invasion, migration, as well as metastasis of hepatocellular cancer cells, cervical cancer cells, breast cancer cells, ovarian cancer cells, as well as colorectal cancer cells [10, 14, 15]. Haplotype studies presented that the CGCC haplotype is related to a reduced risk of CAD, suggesting that the rs619586 AG/GG SNP in MALAT1 acts as a factor preventing CAD. Moreover, MALAT1 is found to work as a prognosis marker in lung adenocarcinoma and squamous cell lung carcinoma [16–18]. The over-expression of MALAT1 is also associated with lymph node metastasis in several forms of cancers. For example, it was discovered that the overexpression of MALAT1 inhibited the metastasis of breast cancer, while the deficiency in MALAT1 expression induced the metastasis of breast cancer. MALAT1 also sequesters TEAD activity [19]. In this study, we performed qPCR to evaluate the expression of KATNB1 mRNA in the tissue samples carrying different genotypes of MALAT1. The expression of KATNB1 mRNA was obviously inhibited in the tissue samples collected from patients with the GGGT genotype. It was also shown that the expression of MALAT1 is correlated to tumor size, the state of lymph node metastasis, as well as the staging of cancer. Abudoureyimu et al. and Zheng et al. also showed that the expression of MALAT1 is substantially upregulated in tissues of PTC [20, 21]. However, clinicopathological analysis revealed no relationship between the expression level of MALAT1 and the clinicopathological properties of PTC [22]. To conclude, MALAT1 can promote the survival and imatinib resistance of CML cells by sponging miR-328 activity, providing an insight regarding the crucial function of MALAT1 as a miRNA sponge in CML [11]. In this study, we evaluated the size of primary cancer, lymph node and brain metastatic lung cancer using. No notable difference was found in the primary lung cancer of patients with different genotypes. The size of lymph node and brain metastatic lung cancer was apparently reduced in patients with the GGGT genotype of MALAT1.

A number of researches have shown that miR-328 expression is dysregulated in several types of cancers to impair the activity of non-metabolic and metabolic pathways [23–25]. In previous research, miR-328 was found in exosomes separated from the tumor draining vein in CC patients while its expression level was related to liver metastasis, indicating that miR-328 can contribute to the disruption of signaling mediated by GLUT1 [26]. Further review of miR-328-3p showed that the down-regulation of miR-328-3p is associated with reduced survival and a higher level of invasion in lymph nodes [27]. In BM positive patients, the raised expression level of miR-328 in both brain and thoracic NSCLC samples suggested the association of miR-328 with the potential of brain metastasis [28].

As the most enriched katanin subunit, katanin P80 (KATNB1) plays a key role in microtubule formation, consequently exerting an effect on nuclear shaping, meiosis, as well as sperm flagellum formation [29, 30]. In a research enrolling 414 breast cancer patients, the relationship between KATNB1 and the clinicopathological features of breast cancer was studied, and the results indicated that the increased expression of KATNB1 was linked to a higher ratio of patients in the N and TNM stages, indicating that the aberrant expression of KATNB1 in breast cancer patients may promote cancer cell metastasis and migration [30–32]. Previous research indicated that katanin P80 is vital in the regulation of microtubule dynamics by binding to nuclear mitotic apparatus proteins and cytoplasmic dynein [30, 33]. In addition, katanin P80 may be involved in the onset of certain cancers including breast cancer as well as prostate cancer [34–36]. In this study, we used luciferase assay to explore the regulatory relationship among MALAT1/miR-328 and KATNB1/miR-328. The luciferase activities of MALAT1 and KATNB1 were significantly inhibited by miR-328 in A549 and H460 cells. In addition, we altered the expression of MALAT1 and miR-328 in A549 and H460 cells. MALAT1 expression was positively correlated with the expression of KATNB1, but negatively correlated with the expression of miR-328 in A549 and H460 cells. With respect to the role of katanin P80 in cancer prognosis, it was only shown that the expression of katanin P80 is valuable in the prediction of worse OS of breast cancer patients, and katanin P80 expression is negatively related to DFS as well as OS [13, 37]. In the subgroup OS analysis, katanin P80-positive breast cancer patients showed worse OS in comparison to katanin P80-negative patients. The main reason is that katanin P80 can affect the migration and proliferation of cells through targeting a number of genes and pathways, such as MACF1 and LAPSER1 genes, consequently increasing the level of lymph node metastasis in breast cancer patients [37].

## CONCLUSION

As a conclusion, our study found that the expression of MALAT1 was reduced in patients carrying haplotype GGGT. A signaling pathway of MALAT1/miR-328/KATNB1 was established in our study, which explained the down-regulated KATNB1 mRNA and katanin P80 levels in patients carrying haplotype GGGT and reduced lymph node size in lung cancer and tumor size in brain metastatic lung cancer.
